# Development and Validation of an In-House Real-Time Reverse-Transcriptase Polymerase Chain Reaction Assay for SARS-CoV-2 Omicron Lineage Subtyping between BA.1 and BA.2

**DOI:** 10.3390/v14081760

**Published:** 2022-08-12

**Authors:** David Pham, Susan Maddocks, Dominic E. Dwyer, Vitali Sintchenko, Jen Kok, Rebecca J. Rockett

**Affiliations:** 1 Centre for Infectious Diseases & Microbiology Laboratory Services, New South Wales Health, Pathology—Institute of Clinical Pathology & Medical Research, Westmead Hospital, Westmead, NSW 2145, Australia; 2 Sydney Infectious Diseases Institute, The University of Sydney, Westmead, NSW 2145, Australia; 3 Centre for Infectious Diseases & Microbiology—Public Health, Westmead Hospital, Westmead, NSW 2145, Australia

**Keywords:** SARS-CoV-2, variants of concern, COVID-19, real-time RT-PCR, Omicron, sublineage

## Abstract

In order to rapidly differentiate sublineages BA.1 and BA.2 of the SARS-CoV-2 variant of concern Omicron, we developed a real-time reverse-transcriptase polymerase chain reaction to target the discriminatory spike protein deletion at amino acid position 69–70 (S:del69–70). Compared to the gold standard of whole genome sequencing, the candidate assay was 100% sensitive and 99.4% specific. Sublineage typing by RT-PCR can provide a rapid, high throughput and cost-effective method to enhance surveillance as well as potentially guiding treatment and infection control decisions.

## 1. Introduction

Severe Acute Respiratory Syndrome Coronavirus 2 (SARS-CoV-2), the cause of Coronavirus Disease 2019 (COVID-19), has been responsible for over half a billion infections and over six million deaths worldwide [1,2]. SARS-CoV-2 is continuously evolving, and several peaks of the pandemic have correlated with the emergence of new virus variants [3]. The World Health Organization (WHO) have identified variants of concern (VOC) and variants of interest (VOI), based on both genetic changes and epidemiological impacts [4].

Variants are monitored in terms of epidemiology, clinical severity, effectiveness of public health or social interventions, diagnostic methods, immune responses, antibody neutralization, vaccine effectiveness or other relevant characteristics [4]. Variants are often further classified into lineages, using a number of internationally recognized schemes. Lineages are defined using phylogenetic analysis or the detection of constellations of mutations using whole genome sequencing (WGS) [5,6]. Most mutations of interest occur in the spike (S) protein, which binds to the angiotensin-converting enzyme 2 (ACE2) cell receptor [7].

Variants are identified primarily by WGS performed by public health laboratories worldwide [8]. Real-time or near-real-time identification of variants can significantly impact the management of the ongoing pandemic, informing individual patient care by guiding appropriate therapy (by the detection of variant-specific antiviral or monoclonal antibody resistance), or public health measures for variants with increased disease severity [9,10,11]. However, local sequencing availability may be limited by access and capacity. Cost, assay throughput, turnaround times and the reduced sensitivity of WGS methods are also limiting factors. These limitations may be overcome by real-time reverse transcriptase polymerase chain reaction (RT-PCR)-based variant analysis of SARS-CoV-2, as an alternative or supplement to WGS [12,13].

In this study, we developed a RT-PCR assay to identify the S protein deletion at amino acid position 69–70 (S:del69–70). This deletion has arisen independently in various lineages, including VOC Alpha and VOI Eta, and most recently in several VOC Omicron sublineages, suggestive of its relevance in convergent evolution of SARS-CoV-2 (Table 1). Laboratory in vitro classification of this deletion shows it is associated with increased infectivity [14]. The use of S:del69–70 as a marker to differentiate or presumptively identify VOC was a serendipitous discovery for VOC Alpha in a SARS-CoV-2 commercial assay (ThermoFisher TaqPath COVID-19) [15]. This assay produces ‘S-gene target failure’ (SGTF) due to its primers or probes overlapping the S protein at positions 69–70 [16]. The Alpha VOC was first observed in the United Kingdom when the incidence of samples with SGTF began to increase dramatically from late November 2020 in three Lighthouse laboratories. Identifying the S:del69–70 mutation has been instrumental in monitoring the epidemiology of circulating variants such as Alpha and Omicron VOC [16].

## 2. Materials and Methods

### 2.1. Population and Samples

Respiratory tract specimens (mostly upper respiratory tract samples, consisting of combined nose and throat swabs, nasopharyngeal swabs or Rhinoswabs (Rhinomed, Richmond, Australia) were collected from patients in either universal or viral transport media. Specimens where SARS-CoV-2 RNA was detected on a validated nucleic acid amplification test (NAAT) are monitored by the New South Wales (NSW) Ministry of Health via the Notifiable Conditions Information Management System (NCIMS). A proportion of these specimens are referred to the state reference public health laboratory for whole genome sequencing. These specimens were collected from, but not limited to, patients admitted to the intensive care unit, recently returned overseas travellers, potential SARS-CoV-2 re-infections, and other patients identified by healthcare providers or public health authorities for WGS. Specimens included in this study were collected from January 2022 to April 2022. 

### 2.2. Extraction of Viral Nucleic Acid

Viral nucleic acid was extracted using the MagNA Pure 96 instrument using the MagNA Pure 96 DNA and Viral NA Small Volume Kit via the Pathogen Universal Protocol (Roche Diagnostics, Mannheim, Germany). Two hundred microlitres of sample was extracted and eluted into 100 µL.

### 2.3. Assay Design

An in-house RT-PCR assay was designed targeting S:del69–70. The 3′ end of the forward primer overlaps the deletion site; the discrepancy in the primer clamp ensures specificity such that wild-type sequences will not be amplified. A previously validated nucleocapsid (N) gene target was chosen to act as an internal control [18]. Primers and probes were obtained from Bioneer Corporation (Daejeon, Republic of Korea) (Table 2).

Four microlitres of total nucleic acid eluate was added to a 16 µL total-volume reaction mixture comprising AgPath-ID One-Step RT-PCR enzyme mix (Thermo Fisher Scientific, Waltham, MA, USA), 500 nM of each primer and 250 nM of each probe. Amplification was carried out on the LightCycler 480 Instrument II (Roche Diagnostics). The reverse transcription phase was performed at 45 °C for 15 min followed by inactivation/denaturation at 95 °C for 15 min. Amplification included 45 cycles of denaturation at 95 °C for 15 s, followed by annealing, extension and data acquisition at 60 °C for 45 s.

Each run included molecular grade nuclease-free water (Thermo Fisher Scientific) as a no-template control, and controls representative of SARS-CoV-2 BA.1 and BA.2 virus were prepared from virus isolates as previously outlined [19]. Briefly, culture supernatant was harvested four days after inoculation. Six hundred microlitres of RNeasy lysis buffer (RNeasy minikit, Qiagen GmbH, Hilden, Germany) was added to 200 µL of culture supernatant and mixed well. An equal volume (800 µL) of 70% ethanol was then added and mixed well by pipetting, before loading onto RNeasy column in successive aliquots until the entire volume was extracted. RNA was eluted in 30 µL and then reverse transcribed using SuperScript IV VILO mastermix (Thermo Fisher Scientific). A 1 × 10^4^ dilution of each cDNA elution was used as the S:del69–70 (BA.1) and S:69–70 wild-type (BA.2) controls. 

### 2.4. Genomic Sequencing

Genome sequencing for SARS-CoV-2 was performed using an Illumina (San Diego, CA, USA) sequencing approach based on the Midnight sequencing protocol using a rebalanced primer pool [20]. Briefly, viral extracts were prepared from respiratory tract samples where SARS-CoV-2 was detected by nucleic acid tests and then reverse transcribed using SuperScript IV VILO mastermix (Thermo Fisher Scientific). 

The viral cDNA was used as input for multiplexed overlapping PCR reactions that span the viral genome using Q5 Hot Start High-Fidelity Master Mix (New England Biolabs, Ipswich, MA, USA). Amplicons were then pooled equally, purified and quantified before Nextera XT library preparation and sequencing with multiplexing on an Illumina NextSeq, iSeq or MiniSeq (150 cycle flow cells) [21]. The raw sequence data was subjected to an in-house quality control procedure prior to further analysis as previously described [22,23]. SARS-CoV-2 genomic lineages were inferred using Phylogenetic Assignment of Named Global Outbreak Lineages (PANGOLIN) (https://github.com/hCoV-2019/pangolin) and NextClade (https://docs.nextstrain.org/projects/nextclade). 

### 2.5. Analytical Performance

The control material cDNA was quantified using a standard curve produced from serial tenfold dilutions of a synthetic SARS-CoV-2 RNA control (Twist Bioscience, San Francisco, CA, USA). Twenty replicates of serial tenfold dilutions of control material were tested to determine the assay’s reliable limit of detection and analytical sensitivity. Analytical specificity (cross-reactivity) of the assay was determined by testing a panel of 50 pathogens which included influenza A, influenza B, parainfluenzaviruses 1–3, respiratory syncytial virus, human metapneumovirus, adenovirus, enterovirus and rhinovirus. Assay precision was determined by testing positive and negative control material in duplicate for 10 runs. Accuracy was evaluated by testing archived specimens which had previously undergone WGS. Clinical sensitivity and specificity were evaluated against WGS as the gold standard, where Sensitivity = [True Positive/(True Positive + False Negative)] × 100% and Specificity = [True Negative/(True Negative + False Positive)] × 100%.

## 3. Results

### 3.1. Analytical Sensitivity

The assay’s reliable limit of detection was determined to be between 1.7–17 copies/µL for both the S:del69–70 and the HKU-N target.

### 3.2. Analytical Specificity

Archived specimens (*n* = 50) known to be positive for influenzavirus A (*n* = 4), influenzavirus B (*n* = 7), parainfluenzavirus 1 (*n* = 5), parainfluenzavirus 2 (*n* = 1), parainfluenzavirus 3 (*n* = 6), respiratory syncytial virus (*n* = 7), human metapneumovirus (*n* = 3), adenovirus (*n* = 5), enterovirus (*n* = 5) and rhinovirus (*n* = 7) were tested against a respiratory multiplex nucleic acid amplification panel as well as the candidate assay. All archived specimens were confirmed positive on the respiratory multiplex panel, and none showed amplification on either target of the candidate assay. 

Thirty-seven consecutive specimens referred for respiratory multiplex nucleic acid amplification were tested against the candidate assay. Of these, three were positive on the candidate assay, and a review of records confirmed that all three samples were collected from patients with known SARS-CoV-2 infection. None of the other 34 specimens showed amplification on either target of the candidate assay.

### 3.3. Reproducibility

Twenty replicates of each control material were tested using all six available LightCycler 480 Instrument II systems at our centre on different days (Table 3).

### 3.4. Accuracy

Four hundred and fifty-six specimens previously referred for WGS were retrospectively tested using the candidate assay. WGS detected 118 BA.1 infections, 266 BA.2 infections, 1 Delta infection and 3 BA.1/BA.2 co-infections but was unsuccessful in assigning lineages for 68 specimens. Using the candidate assay, 148 S:del69–70 (presumptive BA.1) and 290 S:69–70 wild-type (presumptive BA.2) infections were detected. Eighteen specimens failed to produce amplification on either target of the candidate assay (invalid result), most likely secondary to degradation of nucleic acid material in the time between specimen collection and testing.

Discordant results were reviewed. Three specimens where BA.1/BA.2 co-infection was detected by WGS were classified as presumptive BA.1 by the candidate assay. One specimen where Delta infection was detected by WGS was classified as presumptive BA.2 by the candidate assay.

There were three specimens where BA.2 infection was detected by WGS but the candidate assay detected the presence of S:del69–70, resulting in classification as presumptive BA.1. SARS-CoV-2 consensus genomes were reviewed for these three specimens, and one of these specimens was confirmed to be a BA.2 sequence but possessed S:del69–70. The other two discordant results remain unresolved and are possibly due to contamination of archived sample or another pre-analytical error, as the PCR assay result was reproduced when repeated twice. 

Subsequently, 554 consecutive specimens referred for WGS were tested prospectively and in parallel using the candidate assay. Using WGS, 28 BA.1 infections, 428 BA.2 infections and four XE infections were detected. WGS was unsuccessful in assigning lineages for 94 of these specimens. Using the candidate assay, 35 S:del69–70 (presumptive BA.1) and 498 S:69–70 wild-type (presumptive BA.2) were detected. Twenty-one specimens failed to produce amplification on either target of the candidate assay.

Discordant results were reviewed. Four specimens where XE infection was detected by WGS were classified as presumptive BA.2 by the candidate assay. The SARS-CoV-2 consensus genomes was reviewed for one specimen where BA.2 infection was detected by WGS, but the candidate assay identified S:del69–70, resulting in classification as presumptive BA.1. This genome was another BA.2 sequence that possessed S:del69–70.

Compared to the gold standard of WGS for SARS-CoV-2 lineage determination, the sensitivity and specificity of the candidate assay for S:del69–70 was calculated at 100% and 99.4%, respectively (Table 4). The positive predictive value was 97.3% and negative predictive value was 100%. The overall accuracy was 99.5%. Further information is available in the supplementary material.

## 4. Discussion

The surveillance of SARS-CoV-2 variants from the outset of the COVID-19 pandemic has been primarily informed by WGS, but this method is not without its limitations [8]. Its availability is limited to laboratories with next generation sequencing and bioinformatics capabilities. Turnaround times, costs and limited sensitivity also make WGS an impractical tool for identifying VOC in all samples. In NSW, approximately 2% of all SARS-CoV-2 cases confirmed by NAAT have undergone WGS. On the other hand, variant typing by RT-PCR provides a rapid, high throughput and cost-effective method to enhance surveillance as well as potentially guiding antiviral treatment and infection control decisions in a rapid timeframe [24,25]. 

In this study, we demonstrate the development and validation of a rapid and accurate RT-PCR assay targeting the S:del69–70 mutation. Most of the literature regarding the use of S:del69–70 to monitor or type SARS-CoV-2 has been for differentiating Alpha VOC from ancestral SARS-CoV-2, or Delta VOC from Omicron BA.1 VOC. Our assay was evaluated against a population of predominantly Omicron BA.1 and Omicron BA.2. The overall agreement between RT-PCR and WGS in detecting BA.1 and BA.2 variants was >99%. In our centre, WGS requires two working days to deliver a result, while RT-PCR results can be available within six hours. Furthermore, RT-PCR was able to provide a presumptive lineage identification in 76% of specimens that could not be assigned a lineage by WGS. This is most likely due to the greater sensitivity of RT-PCR, as WGS generally requires samples with higher SARS-CoV-2 viral loads (inferred from cycle threshold values below 30) to be successful [26]. Emergence of new variants with single nucleotide polymorphisms (SNPs) in primer binding sites are also more likely to impact WGS than targeted RT-PCR, mostly due to the larger number of primers and increased amplicon size [27]. 

There are multiple places where this candidate assay could fit into the SARS-CoV-2 testing algorithms, depending on local policy or workflow. This assay could be used as an initial test for diagnosis, a screening assay for referred samples to prioritise subsequent WGS, or as an alternative to WGS in rapid presumptive sublineage typing where WGS may be unavailable or unviable in terms of throughput and cost. The candidate assay would be of greatest value discriminating co-circulating variants where a sublineage may possess the deletion while another is wild type—for instance, differentiating Alpha from ancestral SARS-CoV-2, Delta from BA.1, BA.1 from BA.2, and BA.2 from BA.4 or BA.5.

Our assay is cost-efficient to perform compared to both the ThermoFisher TaqPath assay and WGS (approximately AUD 13 vs. AUD 31 vs. AUD 122 per reaction, inclusive of consumables and labour, in our centre). It is noteworthy that there have been significant supply issues for the TaqPath assay in both our jurisdiction and worldwide, so the availability of an alternative assay such as ours may be useful for the wider community in combating COVID-19. Whilst there are other in-house and commercial assays that specifically target the S:del69–70 mutation, usually with probes overlapping the mutation site [28,29,30,31,32,33], our assay is novel in that the primers overlap the mutation site, and it specifically detects for presence of the mutation, rather than ‘failing’ if the mutation is present. Like other in-house developed assays, our assay can also be readily modified to identify novel variants, provided the genomic sequence of the variant is available. Some centres, however, may prefer using a commercial assay due to workflow, simplicity and quality control considerations, as commercial assays are subject to different regulatory requirements and post-marketing surveillance.

There are several limitations to this study. Firstly, the development and validation of the assay occurred during a time when the predominant circulating variants were BA.1 and BA.2. Changes in epidemiology, such as the introduction of new variants or disappearances of certain variants would change the interpretation of the assay and its potential applications. Indeed, the proportion of BA.1 sequences decreased significantly over the study period. The emerging variants BA.4 and BA.5 also possess the S:del69–70 mutation. While this assay has not been fully assessed against BA.4 and BA.5, the first 10 consecutive samples identified as BA.4 or BA.5 by WGS in our centre showed expected amplification of the S:del69–70 target in this RT-PCR. It would not be possible to differentiate BA.1 from BA.4 or BA.5 with this current assay. S:del69–70 can also occur in a proportion of samples belonging to older lineages, such as the Alpha VOC or the Eta VOI, although these are not currently circulating in significant proportion. Likewise, the assay is unable to differentiate lineages which are S:69–70 wild-type, such as Delta and Omicron BA.1, as we noted in our experiment. However, while this assay may not currently serve its original purpose of differentiating BA.1 and BA.2, with current epidemiology, it would be useful in screening patients for BA.2 from BA.4 or BA.5.

There are also limitations to using an RT-PCR approach for genotyping. Given that these assays can only target a select mutation or group of mutations, it cannot achieve the resolution of WGS. SNPs may not be easily detected or targeted by NAAT and phylogenetic trees cannot be constructed. Mixed or recombinant infections may be misclassified, as demonstrated with several samples in this study. Furthermore, the interpretation of NAATs is reliant on knowledge of currently circulating variants. NAATs are also less useful in detecting new variants, mixed or recombinant infections. As new variants emerge, WGS data is required before any new NAATs can be designed to target new mutations. As such, WGS remains a crucial tool in monitoring the evolution of the COVID-19 pandemic, although NAAT genotyping can be a cheap, accurate and rapid supplement, especially where WGS is not available. 

## Figures and Tables

**Table 1:** Proportion of S protein deletion at amino acid position 69–70 in previous and current VOC/VOI. Variants in bold predominantly contain S:del69–70. Worldwide data obtained from CoV-Spectrum.org, analysed on 25 July 2022 [17].

Previous VOC		Previous VOI		Current VOC	
**Alpha**	**99.35%**	Epsilon	1.20%	Omicron (all)	57.02%
Beta	0.05%	Zeta	0.02%	**Omicron BA.1**	**99.33%**
Gamma	0.24%	**Eta**	**98.07%**	Omicron BA.2	0.66%
Delta	0.26%	Theta	0.30%	**Omicron BA.3**	**99.36%**
		Iota	0.09%	**Omicron BA.4**	**99.04%**
		Kappa	0.13%	**Omicron BA.5**	**99.55%**
		Lambda	1.79%		
		Mu	0.18%		

**Table 2:** **A.** Primers and probes for the detection of the S:del69–70 in variants of concern for SARS-CoV-2. **B.** Assay interpretation.

A		
Target	Name	Primer/Probe Sequence (5′-3′)
S:del69–70	S:del69–70_FOR	AATGTTACTTGGTTCCATGTTATCTC *
	S:del69–70_REV	TCTAAAGTAGTACCAAAAATCCAGCC
	S:del69–70_PROBE	HEX-TGCTTCCATTGAGAAGTCTAACA-BHQ2
HKU-N	HKU-N_FOR	TAATCAGACAAGGAACTGATTA
	HKU-N_REV	CGAAGGTGTGACTTCCATG
	HKU-N_PROBE	FAM-GCAAATTGTGCAATTTGCGG-BHQ1
**B**		
**S:del69–70**	**HKU-N**	**Interpretation**
Detected	Detected	S:del69–70 detected (presumptive BA.1)
Detected	Not detected	S:del69–70 detected (presumptive BA.1)
Not detected	Detected	S:del69–70 not detected (presumptive BA.2)
Not detected	Not detected	Invalid result

* Underlined base pairs represents difference from wild-type sequence.

**Table 3:** Control reproducibility (values represent cycle threshold).

	S:del69–70	HKU-N
S:del69–70 (BA.1) control	Mean: 29.58 Range: 28.26–31.49 Standard deviation: 0.89 Coefficient of variation: 3%	Mean: 29.08 Range: 27.40–31.07 Standard deviation: 0.96 Coefficient of variation: 3.3%
S:69–70 wild-type (BA.2) control	Not detected	Mean: 30.33 Range: 28.62–32.77 Standard deviation: 1.35 Coefficient of variation: 4.45%
No-template control	Not detected	Not detected

**Table 4:** Overall assay performance comparison against gold standard assay.

	Gold Standard: WGS					
		BA.1	BA.2	Other	Invalid	Total
Candidate assay: RT-PCR	Presumptive BA.1	146	4 ^1^	3 ^2^	30	183
	Presumptive BA.2	0	690	5 ^3^	93	788
	Invalid	0	0	0	39	39
	Total	146	694	8	162	1010

^1^ Two BA.2 sequences were found to possess the S:del69–70 mutation. ^2^ Three cases of co-infection with BA.1 and BA.2 were found. ^3^ One case of Delta infection and four cases of recombinant XE infection were found.

## Data Availability

Data is contained within the article or supplementary material.

## Supplementary Materials

The following supporting information can be downloaded at: https://www.mdpi.com/article/10.3390/v14081760/s1.
